# Automatic Estimation of Dynamic Lever Arms for a Position and Orientation System

**DOI:** 10.3390/s18124230

**Published:** 2018-12-02

**Authors:** Qiangwen Fu, Sihai Li, Yang Liu, Qi Zhou, Feng Wu

**Affiliations:** 1 School of Automation, Northwestern Polytechnical University, Xi’an 710072, China; lisihai@nwpu.edu.cn (S.L.); liuyang@mail.nwpu.edu.cn (Y.L.); 2 Science and Technology on Aircraft Control Laboratory, FACRI, Xi’an 710065, China; zhouqis@139.com; 3 Shanghai Aerospace Control Technology Institute, Shanghai 201109, China; wufeng_nwpu@163.com

**Keywords:** position and orientation system (POS), lever arms, Kalman filter, error distributions

## Abstract

An inertially stabilized platform (ISP) is generally equipped with a position and orientation system (POS) to isolate attitude disturbances and to focus surveying sensors on interesting targets. However, rotation of the ISP will result in a time-varying lever arm between the measuring center of the inertial measurement unit (IMU) and the phase center of the Global Positioning System (GPS) antenna, making it difficult to measure and provide compensation. To avoid the complexity of manual measurement and improve surveying efficiency, we propose an automatic estimation method for the dynamic lever arm. With the aid of the ISP encoder data, we decompose the variable lever arm into two constant lever arms to be estimated on line. With a complete 21-dimensional state Kalman filter, we accurately and simultaneously accomplish navigation and dynamic lever arm calibration. Our observability analysis provides a valuable insight into the conditions under which the lever arms can be estimated, and we use the error distribution method to reveal which error sources are the most influential. The simulation results demonstrate that the dynamic lever arm can be estimated to within [0.0104; 0.0110; 0.0178] m, an accuracy that is equivalent to the positioning accuracy of Carrier-phase Differential GPS (CDGPS).

## 1. Introduction

Airborne earth observation takes the aircraft as the platform, and it uses remote sensing load such as synthesize aperture radar (SAR) and charge coupled device (CCD) array camera to acquire a wide range, highly accurate and multilayered space-time information of global surface and deep earth [1]. A position and orientation system (POS) is a dedicated strapdown inertial navigation system (SINS)/Global Positioning System (GPS) integrated system for airborne remote sensing [2]. As a direct georeferencing (DG) system, the POS provides the ability to directly relate the data collected by a remote sensing system to earth without using traditional ground-based measurements [3]. To improve mapping accuracy and efficiency, the POS is required to provide the position information to centimeter-level accuracy, and orientation data to sub-arcminute accuracy in either real-time navigation or post-mission, such as the current state-of-the-art airborne system POS AV610 [3,4]. This is primarily accomplished by the inertial measurement unit (IMU) integrated with a Carrier-phase Differential GPS (CDGPS).

The key algorithm for POS is still SINS/GPS integrated navigation [5,6]. For the loosely-coupled integration method discussed here, the positions and velocities of the SINS and GPS solutions are compared, and the resulting differences are used as the measurement values for a Kalman filter [7,8]. Due to the extremely high positioning accuracy of a CDGPS, typically 1–2 cm plus 1 ppm of baseline separation in real-time kinematic (RTK) or post-processing [9,10], many overlooked errors must be treated seriously. Among these, the time and space asynchronies are the dominant error sources. The time asynchrony means the time delay of the GPS position and the velocity information with respect to the SINS data in real-time systems, which will badly influence the accuracy of the SINS/GPS navigation solution. Based on one pulse per second (1PPS) of the GPS, which can deliver very high time accuracies such as 20 ns, the time delay can be significantly attenuated by the professional time synchronization technology [11]. The space asynchrony means the lever arm between the GPS antenna and the IMU. Generally, the GPS antenna is mounted on the roof of the plane for better GPS satellite visibility [7,12], while the IMU is installed inside the plane cabin or under the aircraft belly as close to the surveying sensors as possible to weaken the influence of flexural deformation. Therefore, the phase center of the GPS antenna and the measuring center of the IMU are separated by a lever arm. Since the lever arm in length is usually several meters, it severely deteriorates measurement values under angular excitations if no compensation is provided.

Many efforts have been devoted to improving SINS/GPS integration accuracy by attenuating the lever arm uncertainty. One method is manual measurement by a total station [13,14], which can obtain the length of the lever arm with sufficient accuracy, but suffers from complicated operation. Furthermore, it is a difficult task in itself to determine the phase center of a GPS antenna and the sensing center of an IMU. Repeated measurements are inevitable if the GPS antenna or IMU is replaced or remounted. The other method is automatic identification of the lever arm by the SINS/GPS integration navigation [1,15,16,17,18,19,20], in which the lever arm is extended as an error state of the Kalman filter to be estimated, while the vehicle maneuvers. The latter method is simple and effective, but practical only if the lever arm is constant.

To maintain imaging quality, aerial surveying sensors are required to move in a straight line with a given direction, but a plane may deviate from the ideal motion trajectory, due to gusts, air turbulence, or flight control errors [7,21]. Consequently, an inertially stabilized platform (ISP) with three gimbals is usually used to isolate the angular motion interference and to focus the surveying sensor onto the desired targets, such as the general ISP PAV80 and GSM3000 [7,22,23]. The use of ISP makes the lever arm problem more complicated. Generally, the surveying sensor and the IMU are rigidly mounted close together on the ISP, but the measuring center of the IMU cannot be ensured to coincide with the rotating center of the ISP. When the ISP rotates, the lever arm between the IMU center and the GPS antenna center changes; for this reason, we call it a dynamic lever arm. Since the lever arm is not constant in this situation, traditional lever arm estimation methods do not work. In [7,12], the dynamic lever arm is decomposed into two constant lever arms to be compensated in flying missions, but they are still manually measured in advance. In order to avoid complex manual measurements and to improve surveying efficiency, we propose an automatic approach to estimating two separate lever arms that provide equivalent compensation.

The rest of this paper is organized as follows: Section 2 describes the system model and the problems to be solved. Section 3 presents the proposed integrated navigation approach and analyzes the corresponding observability in detail. Section 4 verifies our method through simulation tests, and conclusions are drawn in Section 5.

## 2. System Description

**Figure 1:**
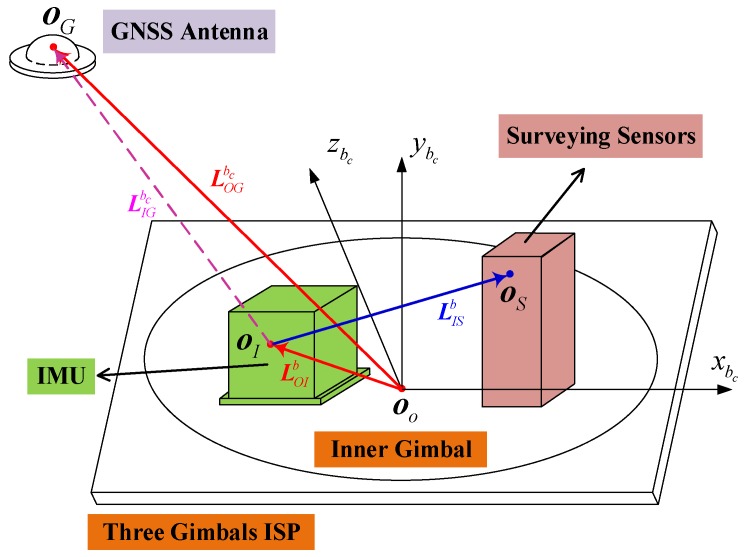
The installation relationship of the position and orientation system (POS) and surveying system.

As shown in Figure 1, the IMU and surveying sensors are usually fixed on the inner gimbal of the ISP. Thus, the mounting angle and lever arm between the two are constant, reducing the accuracy requirements for the ISP. The GPS antenna is mounted on the roof of the plane for better satellite visibility. The related coordinate systems are defined as follows:

n-frame, the navigation frame, chosen as the local level east–north–up (ENU) coordinate;

b-frame, the IMU body frame, implicitly predefined by the calibrated sensitive axes of the inertial sensors, with the origin located at the sensitive center of IMU (point $O_{I}$), and axes pointing right, forward, and upward;

$b_{c}$-frame, the carrier frame, fixed to the flight vehicle and originating at the rotating center of the ISP (point $O_{O}$). The axis directions of $b_{c}$-frame do not rotate with the gimbals, and coincide with the ISP only if the encoder data of three gimbals are all zeros;

$b_{s}$-frame, the surveying sensor frame, rigidly fixed to the surveying sensor with the origin located at the surveying center (point $O_{S}$).

Based on the established frames, the related lever arms are defined as follows:

$L_{OI}^{b}$, the lever arm from the rotating center of the ISP (point $O_{O}$) to the sensitive center of IMU (point $O_{I}$) projected in the b-frame;

$L_{OG}^{b_{c}}$, the lever arm from the rotating center of the ISP (point $O_{O}$) to the phase center of the GPS antenna (point $O_{G}$) projected in the $b_{c}$-frame. Whether the ISP rotates or not, there is no relative motion between the point $O_{O}$ and point $O_{G}$ in the $b_{c}$-frame; thus, the lever arm $L_{OG}^{b_{c}}$ is a constant;

$L_{IG}^{b_{c}}$, the lever arm from the sensitive center of the IMU (point $O_{I}$) to the phase center of the GPS antenna (point $O_{G}$) projected in the $b_{c}$-frame;

$L_{IS}^{b}$, the lever arm from the sensitive center of the IMU (point $O_{I}$) to the measuring center of the surveying center (point $O_{S}$), projected in the b-frame.

Three lever arms—$L_{OI}^{b}$, $L_{OG}^{b_{c}}$, and $L_{IS}^{b}$—are constant, while lever arm $L_{IG}^{b_{c}}$ changes with the ISP rotation. Because the lever arm $L_{IG}^{b_{c}}$ is uncertain and time-varying, it is referred to as a dynamic lever arm.

Since the attitude matrix $C_{b}^{n}$ can be obtained from the SINS solution, the transformation matrix $C_{b_{c}}^{n}$ from $b_{c}$-frame to the n-frame can be obtained as follows:

(1) $$C_{b_{c}}^{n}=C_{b}^{n}C_{b_{c}}^{b},$$

in which the direction cosine matrix $C_{b_{c}}^{b}$ is formed with the encoder data of the ISP [7]:

(2) $$C_{b_{c}}^{b}=C_{\psi_{T}}C_{\theta_{T}}C_{\gamma_{T}},$$

where:

$$C_{\psi_{T}}=\left[\begin{array}{ccc} \cos\psi_{T} & \sin\psi_{T} & 0\\ -\sin\psi_{T} & \cos\psi_{T} & 0\\ 0 & 0 & 1 \end{array}\right],\;C_{\theta_{T}}=\left[\begin{array}{ccc} 1 & 0 & 0\\ 0 & \cos\theta_{T} & \sin\theta_{T}\\ 0 & -\sin\theta_{T} & \cos\theta_{T} \end{array}\right],\;C_{\gamma_{T}}=\left[\begin{array}{ccc} \cos\gamma_{T} & 0 & -\sin\gamma_{T}\\ 0 & 1 & 0\\ \sin\gamma_{T} & 0 & \cos\gamma_{T} \end{array}\right],$$

the angles $\psi_{T}$, $\theta_{T}$, and $\gamma_{T}$ are encoder data obtained from the inner, middle, and outer gimbals of the ISP, respectively.

Referring to Equation (2), the angular rate of the b-frame with respect to the $b_{c}$-frame, denoted as $\omega_{b_{c}b}^{b}$, can be calculated as follows [7]:

(3) $$\omega_{b_{c}b}^{b}=\left[\begin{array}{c} 0\\ 0\\ {\dot{\psi }}_{T} \end{array}\right]+C_{\psi_{T}}\left[\begin{array}{c} {\dot{\theta }}_{T}\\ 0\\ 0 \end{array}\right]+C_{\psi_{T}}C_{\theta_{T}}\left[\begin{array}{c} 0\\ {\dot{\gamma }}_{T}\\ 0 \end{array}\right],$$

where ${\dot{\theta }}_{T}$, ${\dot{\gamma }}_{T}$, and ${\dot{\psi }}_{T}$ are the angular rates of roll, pitch, and heading gimbals of the ISP, respectively, which are obtained by the time differentiation of the corresponding encoder data.

According to the lever arm effect [24], if the position and velocity parameters at point $O_{I}$ are obtained from navigation solutions, and the lever arm $L_{IS}^{b}$ is known, the corresponding information at point $O_{S}$ can be calculated as follows:

(4) $$p_{S}^{}=p_{I}^{}+M_{p}C_{b}^{n}L_{IS}^{b},$$

(5) $$v_{S}^{n}=v_{I}^{n}+C_{b}^{n}\left(\omega_{eb}^{b}\times L_{IS}^{b}\right),$$

(6) $$M_{p}=\left[\begin{array}{ccc} 0 & 1/\left(R_{M}+h\right) & 0\\ \sec L/\left(R_{N}+h\right) & 0 & 0\\ 0 & 0 & 1 \end{array}\right],$$

where $p_{S}^{}$ and $p_{I}^{}$ denote the position information consisting of latitude, longitude, altitude; $v_{S}^{n}$ and $v_{I}^{n}$ are the velocity data at point $O_{S}$ and point $O_{I}$ in the n-frame; L and h are the local latitude and height; $R_{N}$ and $R_{M}$ denote the transverse and meridian radius of curvature. The relative angular rate $\omega_{eb}^{b}$ is obtained by $\omega_{eb}^{b}=\omega_{ib}^{b}-C_{n}^{b}\omega_{ie}^{n}$, in which $\omega_{ib}^{b}$ can be acquired from the output of gyros, and $\omega_{ie}^{n}$ denotes earth’s rotation rate vector in the n-frame.

Similarly, the position and velocity parameter correlations among the point $O_{O}$, point $O_{I}$, and point $O_{G}$ are formulated as:

(7) $$\left\{ \begin{array}{l} p_{I}^{}=p_{O}^{}+M_{p}C_{b}^{n}L_{OI}^{b}\\ v_{I}^{n}=v_{O}^{n}+C_{b}^{n}\left(\omega_{eb}^{b}\times L_{OI}^{b}\right) \end{array}\right.$$

(8) $$\left\{ \begin{array}{l} p_{G}^{}=p_{O}^{}+M_{p}C_{b_{c}}^{n}L_{OG}^{b_{c}}\\ v_{G}^{n}=v_{O}^{n}+C_{b_{c}}^{n}\left(\omega_{eb_{c}}^{b_{c}}\times L_{OG}^{b_{c}}\right) \end{array}\right.$$

(9) $$\left\{ \begin{array}{l} p_{G}^{}=p_{I}^{}+M_{p}C_{b_{c}}^{n}L_{IG}^{b_{c}}\\ v_{G}^{n}=v_{I}^{n}+C_{b_{c}}^{n}\left(\omega_{eb_{c}}^{b_{c}}\times L_{IG}^{b_{c}}\right) \end{array}\right.$$

where $v_{O}^{n}$ and $v_{G}^{n}$ denote the velocities at point $O_{O}$ and point $O_{G}$ in the n-frame, and the relative angular rate $\omega_{eb_{c}}^{b_{c}}$ can be obtained by:

(10) $$\omega_{eb_{c}}^{b_{c}}=C_{b}^{b_{c}}\left(\omega_{eb}^{b}-\omega_{b_{c}b}^{b}\right)$$

In a normal integrated navigation mechanism, the velocity and/or position differences between the SINS and GPS solutions are generally taken as the measurement values. To improve the measurement accuracy, the dynamic lever arm $L_{IG}^{b_{c}}$ must be compensated precisely even if it is difficult. Based on Equations (7) and (8), the dynamic lever arm can be compensated as follows:

(11) $$\left\{ \begin{array}{l} p_{I}^{}-p_{G}^{}=-M_{p}C_{b_{c}}^{n}\left(L_{OG}^{b_{c}}-C_{b}^{b_{c}}L_{OI}^{b}\right)\\ v_{I}^{n}-v_{G}^{n}=-C_{b_{c}}^{n}\left[\omega_{eb_{c}}^{b_{c}}\times \left(L_{OG}^{b_{c}}-C_{b}^{b_{c}}L_{OI}^{b}\right)\right] \end{array}\right.$$

Combining Equations (9) and (11) yields:

(12) $$L_{IG}^{b_{c}}=L_{OG}^{b_{c}}-C_{b}^{b_{c}}L_{OI}^{b}.$$

This means that the dynamic lever arm $L_{IG}^{b_{c}}$ can be decomposed into two constant lever arms $L_{OG}^{b_{c}}$ and $L_{OI}^{b}$, because of the fact that the ISP rotating center $O_{O}$ is fixed to both the b-frame and the $b_{c}$-frame. From the geometric location relationship shown in Figure 1, we can deduce the same results as Equation (12). Therefore, if the two constant lever arms $L_{OG}^{b_{c}}$ and $L_{OI}^{b}$ are precisely measured in advance, the dynamic lever arm $L_{IG}^{b_{c}}$ can be compensated as Equation (12). However, as mentioned before, manual measurement is complex, tedious, and inefficient. To avoid these drawbacks and enhance the efficiency of surveying, we propose a method to automatically estimate the lever arm and to accomplish the integrated navigation simultaneously.

## 3. Proposed SINS/GPS Integrated Navigation Model

### 3.1. SINS/GPS Integrated Navigation Model

As shown in Equation (11), if the SINS/GPS integrated navigation model can be designed to estimate the lever arms $L_{OG}^{b_{c}}$ and $L_{OI}^{b}$, the complex dynamic lever arm $L_{IG}^{b_{c}}$ can be compensated automatically, and the tedious work of manual measurement can be eliminated. Combining the classical error model of SINS and the constant lever arms $L_{OI}^{b}$ and $L_{OG}^{b_{c}}$, we define a 21-dimensional error state vector of SINS/GPS integration as:

(13) $$x\left(t\right)={\left[\begin{array}{ccccccc} {\left(\phi^{n}\right)}^{T} & {\left(\delta v_{}^{n}\right)}^{T} & {\left(\delta p_{I}\right)}^{T} & {\left(\varepsilon^{b}\right)}^{T} & {\left(\nabla^{b}\right)}^{T} & {\left(L_{OI}^{b}\right)}^{T} & {\left(L_{OG}^{b_{c}}\right)}^{T} \end{array}\right]}^{T},$$

where $\phi^{n}=[\phi_{E},\phi_{N},\phi_{U}{]}^{T}$ is the misalignment angles of SINS; $\delta v_{}^{n}\Pi =[\delta v_{E},\delta v_{N},\delta v_{U}{]}^{T}$ is the velocity errors; $\delta p_{I}={\left[\delta L,\delta \lambda ,\delta h\right]}^{T}$ denotes the position errors consisting of latitude, longitude, and height errors; $\varepsilon^{b}$ and $\nabla^{b}$ are the gyro and accelerometer biases in the b-frame, respectively.

The corresponding state equation of Kalman filter is:

(14) $$\dot{x}\left(t\right)=\left[\begin{array}{cc} F_{I} & 0_{15\times 6}\\ 0_{6\times 15} & 0_{6\times 6} \end{array}\right]x\left(t\right)+\left[\begin{array}{c} -C_{b}^{n}\varepsilon_{w}^{b}\\ {{}_{}}_{}C_{b}^{n}\nabla_{w}^{b}\\ 0_{15\times 1} \end{array}\right],$$

where $F_{I}$ is a 15 × 15 transition matrix based on typical SINS error model as detailed in [25]. The symbols $\varepsilon_{w}^{b}$ and $\nabla_{w}^{b}$ are the noises of gyros and accelerometers, and $0_{i\times j}$ denotes an i×j zero matrix.

For loosely coupled SINS/GPS integrated navigation, there are two measurement models to be selected in practical POS, including position measurements and position/velocity measurements. The associated measurement equations are:

(15) $$\left\{ \begin{array}{l} z_{p}=p_{I}^{}-p_{G}^{}=H_{p}x\left(t\right)+w_{gp}\\ z_{v}=v_{I}^{n}-v_{G}^{n}=H_{v}x\left(t\right)+w_{gv} \end{array}\right.,$$

where $w_{gp}$ and $w_{gv}$ represent the position and velocity noises of the GPS.

Since Equation (11) can be rewritten as:

(16) $$\left\{ \begin{array}{l} p_{I}^{}-p_{G}^{}=M_{p}C_{b}^{n}L_{OI}^{b}-M_{p}C_{b_{c}}^{n}L_{OG}^{b_{c}}\\ v_{I}^{n}-v_{G}^{n}=C_{b}^{n}\left(\omega_{eb}^{b}\times L_{OI}^{b}\right)-C_{b_{c}}^{n}\left(\omega_{eb_{c}}^{b_{c}}\times L_{OG}^{b_{c}}\right) \end{array}\right.,$$

the measurement matrices $H_{p}$ and $H_{v}$ in Equation (15) are obtained as:

(17) $$\left\{ \begin{array}{l} H_{p}=\left[\begin{array}{ccccccc} 0_{3\times 3} & 0_{3\times 3} & I_{3} & 0_{3\times 3} & 0_{3\times 3} & M_{p}C_{b}^{n} & -\;M_{p}C_{b}^{n}C_{b_{c}}^{b} \end{array}\right]\\ H_{v}=\left[\begin{array}{ccccccc} 0_{3\times 3} & I_{3\times 3} & 0_{3\times 3} & 0_{3\times 3} & 0_{3\times 3} & C_{b}^{n}\left(\omega_{eb}^{b}\times \right) & -\;C_{b_{c}}^{n}\left(\omega_{eb_{c}}^{b_{c}}\times \right) \end{array}\right] \end{array}\right..$$

In this paper, we depict the position measurement equation as an example, while the position/velocity measurement equations have the same results.

### 3.2. Observability Analysis for Lever Arms

Usually, an observability analysis for an integrated navigation system is conducted to determine whether a specific error state is observable, and under what conditions it can be estimated [26]. According to global observability theory [27], a state is said to be observable if it can be determined uniquely from the measurements for a finite time and under a possible condition. Therefore, we investigate the observability of lever arms directly, depending on the measurement equation in this section.

The position measurement in Equation (15) can be rewritten as an error model:

(18) $$z_{p}=\delta p_{I}-M_{P}C_{b_{c}}^{n}\left(L_{OG}^{b_{c}}-C_{b}^{b_{c}}L_{OI}^{b}\right)+w_{gp}.$$

Clearly, this is not a deterministic system, but a stochastic case. However, the related theoretical analysis is still useful for revealing the observable conditions and guiding the design of verification trajectories. Ignoring the driving noise and measurement noise temporarily, Equation (18) itself determines the observability of the associated errors $\delta p_{I}$, $L_{OI}^{b}$, and $L_{OG}^{b_{c}}$. It can be seen that the acceleration or deceleration excitations have little effect on lever arms $L_{OI}^{b}$ and $L_{OG}^{b_{c}}$, and their observabilities are determined by direction cosine matrices $C_{b_{c}}^{n}$ and $C_{b}^{b_{c}}$.

If only the ISP rotates, $C_{b}^{b_{c}}$ changes while $C_{b_{c}}^{n}$ remains constant; thus, the lever arm $L_{OI}^{b}$ will be distinguished. In this situation, the SINS position error cannot be accurately estimated for the unobserved lever arm $L_{OG}^{b_{c}}$. Considering the construction of attitude matrix $C_{b}^{b_{c}}$, the rotation of one ISP gimbal can reveal two components of the lever arm $L_{OI}^{b}$ perpendicular to the rotating axis. Consequently, rotations of at least two gimbals are needed to estimate all three components. 

If only the attitude of the vehicle changes, $C_{b_{c}}^{n}$ varies while $C_{b}^{b_{c}}$ stays the same, and the combined lever arm $L_{IG}^{b_{c}}=L_{OG}^{b_{c}}-C_{b}^{b_{c}}L_{OI}^{b}$ can be estimated. Thus, position measurement accuracy can be improved in spite of the unseparated lever arm $L_{OI}^{b}$. It can be seen that the change in $C_{b_{c}}^{n}$ is crucial to determining the final positioning accuracies. Similarly, improvement of all three dimensional position accuracies depends on attitude changing in at least two directions of the vehicle.

In summary, the estimate of constant lever arms $L_{OI}^{b}$ and $L_{OG}^{b_{c}}$ requires the change of direction cosine matrices $C_{b}^{b_{c}}$ and $C_{b_{c}}^{n}$. The greater the change of the matrixes, the stronger the observability of the corresponding lever arms. Certainly, the final estimation accuracies of associated lever arms are limited by the measurement error noise $w_{gp}$.

In aerial mapping missions, the aircraft usually maneuvers intentionally before entering the sensing area to enhance the observability of the POS [17,20], especially to improve the heading accuracy. During the mapping process, long straight portions of the flight will cause heading error growth; thus, periodical aircraft maneuvers are required to separate the navigation errors from inertial sensor errors [18]. Common flight maneuvers include circling, coordinated turn, or “8” shaped flight, in which at least two directions of attitude change occur, such as in the heading and roll axes. Thus, in those cases without ISP, the lever arm $L_{IG}^{b_{c}}$ is usually treated as a constant to be estimated in these planned maneuver processes, and satisfactory results can be obtained [1,17]. As long as any two gimbals of ISP are controlled to rotate during these auxiliary maneuvering processes, the two constant lever arms $L_{OI}^{b}$ and $L_{OG}^{b_{c}}$ are solvable. Therefore, the observable conditions for the dynamic lever arm estimation are easy to satisfy in practical applications.

It should be noted that the auxiliary matrix $C_{b}^{b_{c}}$ is constructed from the encoder data of the ISP, as shown in Equation (2). Since there are some unavoidable errors in the encoder angle measurements, their influences should be evaluated. Denoting the angle measurement errors as $\mu^{b_{c}}$, the introduced position errors are deduced as follows:

(19) $$\delta p_{\mu }=-M_{P}C_{b}^{n}C_{b_{c}}^{b}\delta C_{b_{c}}^{b}L_{OG}^{b_{c}}=M_{P}C_{b_{c}}^{n}(L_{OG}^{b_{c}}\times \mu^{b_{c}}).$$

The term $\left(L_{OG}^{b_{c}}\times \mu^{b_{c}}\right)$ determines the magnitude of the position errors that are introduced by encoder errors. Assuming that the lever arm $L_{OG}^{b_{c}}$ is 2 m and the encoder angle errors of the ISP are 3 arcminutes, the related position errors will be at the 2 mm level. The commonly used ISP PAV80 [22] and GSM3000 [23] can provide such angular accuracy. In this situation, the influence of encoder angle errors is limited and much smaller than the positioning accuracy of typical CDGPS. However, if the lever arm $L_{OG}^{b_{c}}$ is too large, the interrelated influence will not be negligible. The residual encoder error of the ISP can be modelled as a zero mean white noises process, which has the same effect on automatic estimation method and manual measurement method of lever arm. Thus, the GPS antenna and the ISP should be installed as close as possible, otherwise higher performance ISP is needed.

The time delay of the GPS information relative to SINS is another major error source. Since the GPS receiver and the SINS unit are two separate subsystems, the frequency difference between the 1PPS and SINS sampling clock could cause the time delay to be a variable (usually a ramp or triangular wave). Thus, the method of taking the time delay as a constant error state to be estimated will not work. The effective way is to discipline the SINS sampling clock with 1PPS [28], or to record the delayed time with a counter for software compensation [11]. Even so, it is difficult for the time delay to be perfectly compensated. In addition, although the same sampling clock is used in the IMU, the time-asynchrony between each inertial sensor is inevitable, due to the differences of physical characteristics [29], which makes the time synchronization more complicated. Denoting the time-asynchrony error as δt, its influence on position measurement is:

(20) $$\delta p_{dt}=M_{P}v_{}^{n}\delta t.$$

Assuming that δt is 0.1 ms and the normal flight velocity is 50 m/s, the introduced position error is about 5 mm. Clearly, larger time-asynchrony error is intolerable at this speed. After time compensation as mentioned earlier, the residual time-asynchrony error can be modelled as a zero mean white noises process for analysis.

### 3.3. Error Distribution Analysis

The purpose of error distribution analysis for stochastic systems is to evaluate the final estimation accuracy of the concerned states, and to distinguish which error sources primarily contribute to the concerned estimation uncertainties. Monte-Carlo simulations are appropriate to determine error distributions, but suffer from a large computational burden. Based on the law of large numbers [30], Monte-Carlo simulations necessitate a large number of single-factor simulation tests and very long times to establish the error distributions if many error sources are involved. Considering linear error propagation, covariance simulation programs [31,32] are commonly used to provide numerical time histories depicting the accuracy of a given system configuration in terms of the covariance of its associated linearized error state vector, avoiding large numbers of simulations. According to the established SINS/GPS Kalman filter, we designed a covariance analysis method to get the error distribution budgets, especially focusing on which error sources contribute to the estimation uncertainty of lever arms and the final position errors.

Three system models are involved in the covariance analysis method: the real world model, the full-order design filter, and the reduced-order filter model [33]. The real world model, also known as true model, describes the behavior of the actual system, to the best of one’s knowledge and ability. The full-order filter is dedicated to yielding the estimate for all state errors of the real world. The reduced-order filter model is designed for filtering calculation in actual system, in which some weakly observable states have been excluded from the full-order filter to ease computational burden.

In this paper, the real world model is defined in Appendix A, the corresponding full-order filter is depicted in Appendix B, and the reduced-order filter model is designed in Section 3.1. The state vector of the reduced-order filter is composed of the first 21 elements of the full-order filter state. In addition, the full-order filter model contains three types of measurement noise, the GPS positioning noise, the ISP encoder noise and the time-asynchrony noise, and only the first type is selected as the measurement noise of the reduced-order filter model.

For the reduced-order filter model, after the state equation and measurement equation are established and discretized, we update the standard linear Kalman model in two calculation loops [34]: the state filtering loop and covariance calculation loop. For ease of depiction, the discretized state filtering loop is listed here:

(21) $${\hat{x}}_{k}=\Phi_{k,k-1}{\hat{x}}_{k-1},$$

(22) $${\hat{x}}_{k}={\hat{x}}_{k/k-1}+{\hat{K}}_{k}\left({\hat{z}}_{k}-{\hat{H}}_{k}{\hat{x}}_{k/k-1}\right),$$

while the covariance calculation loop is updated as follows:

(23) $${\hat{P}}_{k/k-1}={\hat{\Phi }}_{k,k-1}{\hat{P}}_{k-1}{\hat{\Phi }}_{k,k-1}^{T}+{\hat{\Gamma }}_{k-1}{\hat{Q}}_{k-1}{\hat{\Gamma }}_{k-1}^{T},$$

(24) $${\hat{K}}_{k}={\hat{P}}_{k/k-1}{\hat{H}}_{k}^{T}{\left({\hat{H}}_{k}{\hat{P}}_{k/k-1}{\hat{H}}_{k}^{T}+{\hat{R}}_{k}\right)}^{-1},$$

(25) $${\hat{P}}_{k}=\left(I-{\hat{K}}_{k}{\hat{H}}_{k}\right){\hat{P}}_{k/k-1}{\left(I-{\hat{K}}_{k}{\hat{H}}_{k}\right)}^{T}+{\hat{K}}_{k}{\hat{R}}_{k}{\hat{K}}_{k}^{T},$$

where the hat label “^” denotes the calculated value in the reduced-order filter model, distinguishing it from that of the full-order filter model; the subscript k−1 and k denote the last and current cycle, while k/k−1 expresses the propagation value; ${\hat{\Phi }}_{k,k-1}$, ${\hat{\Gamma }}_{k-1}$ and ${\hat{H}}_{k}$ are discretized values from the state equation and measurement equation; ${\hat{Q}}_{k-1}$ and ${\hat{R}}_{k}$ are the previously set covariance values of process noises and measurement noises; ${\hat{P}}_{k}$ is the current covariance matrix recursively calculated from the initial value ${\hat{P}}_{0}$; and ${\hat{K}}_{k}$ is the calculated filtering gain matrix.

It can be seen that the covariance calculation loop is independent of the state filtering loop. Once the filter is designed and the trajectory is selected, the associated matrices ${\hat{\Phi }}_{k,k-1}$, ${\hat{P}}_{0}$, ${\hat{Q}}_{k-1}$, ${\hat{R}}_{k}$ and ${\hat{H}}_{k}$ are uniquely determined, so is the gain matrix ${\hat{K}}_{k}$. The filtering program only needs to be run once, so that the gain matrix ${\hat{K}}_{k}$ can be obtained at every measurement update. It should be recorded and substituted into the full-order filter model for covariance analysis.

In the full-order filter model, the state equation and measurement equation are extended as follows:

(26) $$x_{k}^{*}=\phi_{k/k-1}^{*}x_{k-1}^{*}+\Gamma_{k-1}^{*}W_{k-1}^{*}$$

(27) $$z_{k}^{*}=H_{k}^{*}x_{k}^{*}+V_{k}^{*}$$

where the superscript “*” denotes the matrix involved in the full-order filter model.

It can be seen that there are three types of errors that affect the estimation results: the initial state errors $x_{0}^{*}$, the process noises $W_{k-1}^{*}$, and the measurement noises $V_{k}^{*}$. Based on the linear error model, the estimated error of a concerned state at any moment can be expressed as a linear cumulative sum:

(28) $$x_{i}^{*}=\sum_{j=1}^{n}S_{i,j}^{*}x_{j0}^{*}+\sum_{j=1}^{l}x_{i,W_{j}}^{*}+\sum_{j=1}^{m}x_{i,V_{j}}^{*},$$

where $S^{*}$ is the propagation matrix of initial errors; n, l, and m are the nonzero element numbers of the initial state, process noise, and measurement noise, respectively; $x_{i,W_{j}}^{*}$ or $x_{i,V_{j}}^{*}$ denotes the estimated error of ith state caused by the jth process noise $W_{j}$ or the jth measurement noise $V_{j}$. For the sake of simplicity, the current cycle k is omitted in Equation (28).

The filtering gain matrix ${\hat{K}}_{k}$ is the crucial link between the reduced-order filter model and the full-order filter model, which can be extended into the full-order filter model as follows:

(29) $$K_{k}^{*}=\left[\begin{array}{c} {\hat{K}}_{k}\\ 0_{18\times 3} \end{array}\right].$$

Since the gain matrix $K_{k}^{*}$ of full-order filter model is known, the covariance calculation loop is updated as follows:

(30) $$P_{k/k-1}^{*}=\Phi_{k/k-1}^{*}P_{k-1}^{*}\Phi_{k/k-1}^{*T}+\Gamma_{k-1}^{*}Q_{k-1}^{*}\Gamma_{k-1}^{*T},$$

(31) $$P_{k}^{*}=\left(I-K_{k}^{*}H_{k}^{*}\right)P_{k/k-1}^{*}{\left(I-K_{k}^{*}H_{k}^{*}\right)}^{T}+K_{k}^{*}R_{k}^{*}K_{k}^{*T}.$$

Firstly, based on linear propagation characteristics of associated errors, and setting $Q_{}^{*}=0$ and $R_{}^{*}=0$ in Equations (30) and (31), the mean square deviation propagation matrix of initial errors can be calculated as follows:

(32) $$S_{k}^{*}=\left(I-K_{k}^{*}H_{k}^{*}\right)\Phi_{k/k-1}^{*}S_{k-1}^{*},$$

with the initial value $S_{0}^{*}=I$.

Secondly, setting $P_{0}^{*}=0$ and $R_{}^{*}=0$ in Equations (30) and (31), the propagation matrix of the jth process noise $W_{j}$ is obtained by:

(33) $$A_{k/k-1}^{j}=\Phi_{k/k-1}^{*}A_{k-1}^{j}\Phi_{k/k-1}^{*T}+\Gamma_{k-1}^{*}e_{jj,k-1}^{}\Gamma_{k-1}^{*T},$$

(34) $$A_{k}^{j}=\left(I-K_{k}^{*}H_{k}^{*}\right)A_{k/k-1}^{j}{\left(I-K_{k}^{*}H_{k}^{*}\right)}^{T},$$

with the initial value $A_{0}^{j}=0$, where $e_{jj}^{}$ denotes the 39 × 39 matrix with e(j,j)=1 and all else zeros. If there are l-dimensional nonzero process noises, Equations (33) and (34) should be executed l times with different $e_{jj}^{}$ to obtain all of the propagation matrices.

Thirdly, setting $P_{0}^{*}=0$ and $Q_{}^{*}=0$ in Equations (30) and (31), the propagation matrix of the jth measurement noise $V_{j}$ is obtained by:

(35) $$B_{k/k-1}^{j}=\Phi_{k/k-1}^{*}B_{k-1}^{j}\Phi_{k/k-1}^{*T},$$

(36) $$B_{k}^{j}=\left(I-K_{k}^{*}H_{k}^{*}\right)B_{k/k-1}^{j}{\left(I-K_{k}^{*}H_{k}^{*}\right)}^{T}+K_{k}^{*}e_{jj,k}^{}K_{k}^{*T},$$

with the initial value $B_{0}^{j}=0$ and m calculations required for m-dimensional measurement noises.

Therefore, the estimation accuracy of $x_{i}^{*}$ in Equation (28) can be evaluated by the corresponding mean square deviation $\sigma_{xi}$:

(37) $$\sigma_{xi}=\sqrt{\sum_{j=1}^{n}S_{i,j}^{2}\sigma_{x_{j0}^{*}}^{2}+\sum_{j=1}^{l}A_{ii}^{j}Q_{jj}^{}+\sum_{j=1}^{m}B_{ii}^{j}R_{jj}^{}},$$

where $\sigma_{x_{j0}^{*}}^{2}$, $Q_{jj}^{}$, and $R_{jj}^{}$ are the deviations of the jth initial state error, jth process noise, and jth measurement noise, respectively, while $A_{ii}^{j}$ and $B_{ii}^{j}$ are the ith diagonal elements of covariance matrices $A_{k}^{j}$ and $B_{k}^{j}$, respectively. Based on the linear error model, the mean square deviation $\sigma_{xi,xj}$ of linear combination of $x_{i}^{*}$ and $x_{j}^{*}$ can be expressed as $\sqrt{\sigma_{xi}^{2}+\sigma_{xj}^{2}}$, and so on.

Therefore, according to the above covariance analyzing method, the error distribution budgets of the full order filter or real-world model can be constructed from the calculation of the reduced order filter. The complexity and computational burden of simulation are greatly reduced.

## 4. Simulation Analysis

### 4.1. Simulation Conditions

Considering the extremely high accuracy requirements for a lever arm in POS, it is difficult to find another more accurate benchmark to evaluate the estimation results. As an expedient, we carried out a series of simulation tests instead of real tests to verify the proposed method. Considering the effect of the time-asynchrony error, we assume that the aircraft flies at 50 m/s uniform speed, and the angular motion excitations are added into the simulation trajectory. The initial values needed for the trajectory are given as:

- Initial position: latitude, longitude and altitude [34.2°; 108.9°; 400 m];
- Initial Velocity: $v_{0}^{n}$ = [0; 50; 0] m/s;
- Initial Attitude: pitch, roll and heading [0; 0; 0]°.
- Lever arms to be estimated: $L_{OI}^{b}$ = [−0.3; 0.1; 0.2] m, $L_{OG}^{b_{c}}$ = [0.5; −2.0; 2.0] m.

To verify the observability analysis results in Section 3.2, two simulation trajectories with different rotation sequences are designed as shown in Table 1. The total time of one simulation trajectory is 150 s, and the related rotation angular rates in two trajectories are all set to 10°/s.

**Figure 2:**
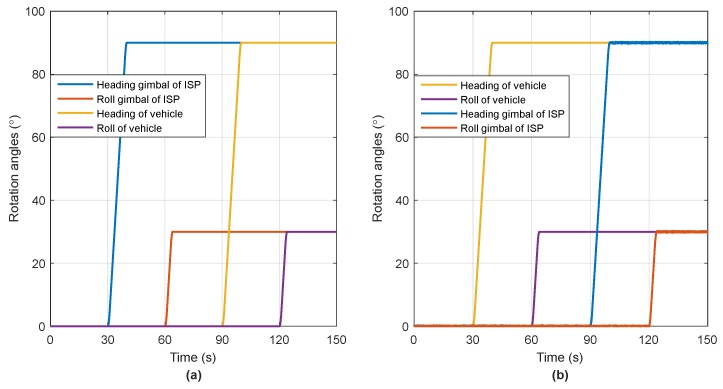
The simulated angular motion trajectories and (**a**) Trajectory 1; (**b**) Trajectory 2.

As shown in Table 1, the only difference between the two trajectories is that steps 2 and 3 are exchanged, which means that the change of matrices $C_{b}^{b_{c}}$ and $C_{b_{c}}^{n}$ occur in a different time sequence. The rotation angles of ISP gimbals and vehicular attitudes are shown in Figure 2.

Referring to the system POS AV610 produced by Applanix [4,35], we simulated an aviation-grade SINS and a CDGPS receiver with sensor specifications, including initial errors, process noises, and measurement noises listed in Table 2. Without loss of generality, the IMU data is sampled at 100 Hz, the ISP encoder data outputs at 20 Hz, and the GPS positioning information at 1 Hz. 

### 4.2. Simulation Results of Lever Arm Estimation

**Figure 3:**
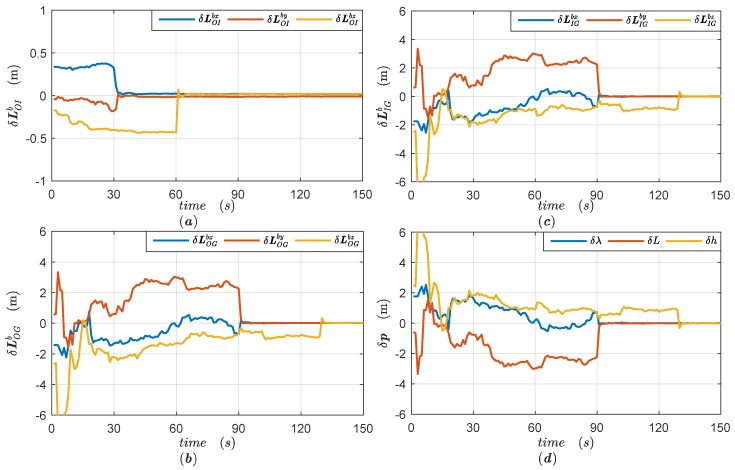
The simulation results of Trajectory 1 and (**a**) estimation errors of the lever arm $L_{OI}^{b}$; (**b**) estimation errors of the lever arm $L_{OG}^{b_{c}}$; (**c**) estimation errors of the combined dynamic lever arm $L_{IG}^{b}$; (**d**) position errors of strapdown inertial navigation system (SINS)/Global Positioning System (GPS) integrated navigation.

**Figure 4:**
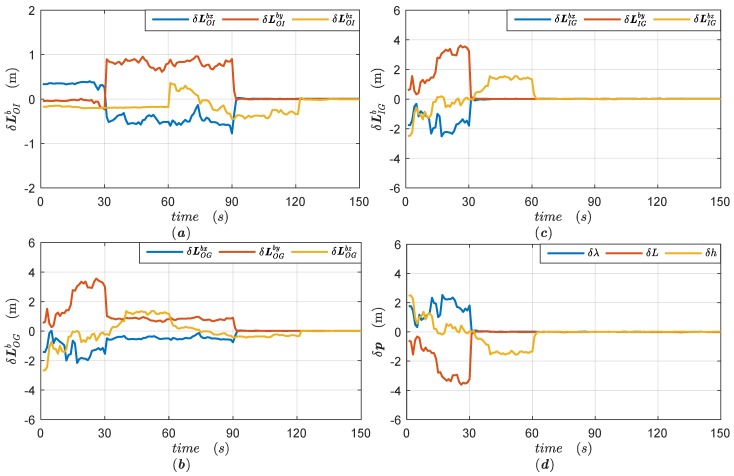
The simulation results of Trajectory 2 and (**a**) estimation errors of the lever arm $L_{OI}^{b}$; (**b**) estimation errors of the lever arm $L_{OG}^{b_{c}}$; (**c**) estimation errors of the combined dynamic lever arm $L_{IG}^{b}$; (**d**) position errors of SINS/GPS integrated navigation.

We use the reduced-order filter model with parameters set as in Appendix C to estimate the lever arms and position errors in two different trajectories. The simulation results of Trajectory 1 are illustrated in Figure 3, while the simulation results of Trajectory 2 are given in Figure 4.

Figure 3a shows the estimation errors of lever arm $L_{OI}^{b}$, Figure 3b,c illustrate the estimation errors of lever arm $L_{OG}^{b_{c}}$ and dynamic lever arm $L_{IG}^{b_{c}}$, while Figure 3d gives the position errors of SINS/GPS integrated navigation. As shown in Figure 3a, the heading gimbal of ISP rotates by 90° at 30 s, and the x and y components of lever arm $L_{OI}^{b}$ are estimated correctly. When the roll gimbal of the ISP rotates by 30° at 60 s, the remaining component $L_{OI}^{bz}$ is identified quickly. It can be seen that the rotations of ISP gimbals can distinguish the lever arm $L_{OI}^{b}$, but have no effect on lever arm $L_{OG}^{b_{c}}$. Therefore, there are still significant position errors in integrated navigation before 90 s as shown in Figure 3d. Once the vehicular attitude changes at 90 s and 120 s, the combined lever arm $L_{IG}^{b_{c}}=L_{OG}^{b_{c}}-C_{b}^{b_{c}}L_{OI}^{b}$ is estimated as in Figure 3c and the position errors converge immediately as shown in Figure 3d. Since the lever arm $L_{OI}^{b}$ is identified, the constant lever arm $L_{OG}^{b_{c}}$ is determined with the convergence of lever arm $L_{IG}^{b_{c}}$.

As shown in Figure 4c, the combined lever arm $L_{IG}^{b}$ is identified when the vehicular heading turns at 30 s and the roll rotates at 60 s. Clearly, the change of attitude matrix $C_{b_{c}}^{n}$ is helpful to estimate the equivalent lever arm $L_{IG}^{b}$. Even though the constant lever arms $L_{OI}^{b}$ and $L_{OG}^{b_{c}}$ cannot be separated, it does not affect the positioning accuracy of SINS/GPS, as shown in Figure 4d. As the ISP gimbals begin to rotate at 90 s and 120 s, the lever arms $L_{OI}^{b}$ and $L_{OG}^{b_{c}}$ are further separated, as shown in Figure 4a,b.

**Figure 5:**
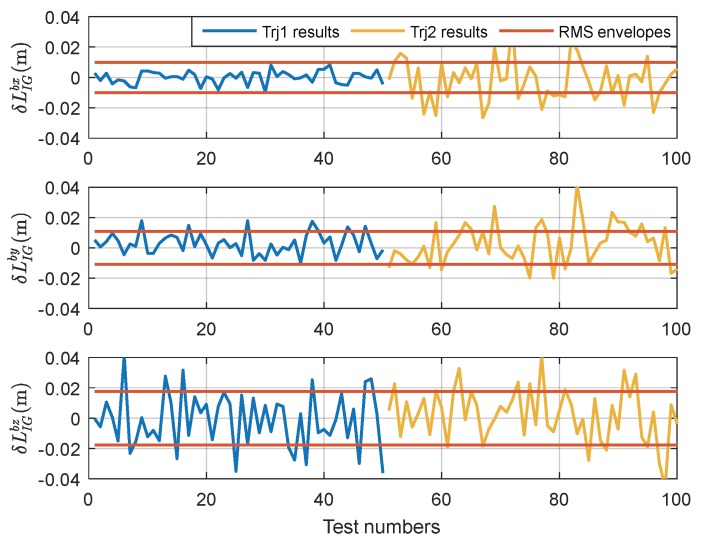
The estimation results of dynamic lever arm $L_{IG}^{b}$.

**Figure 6:**
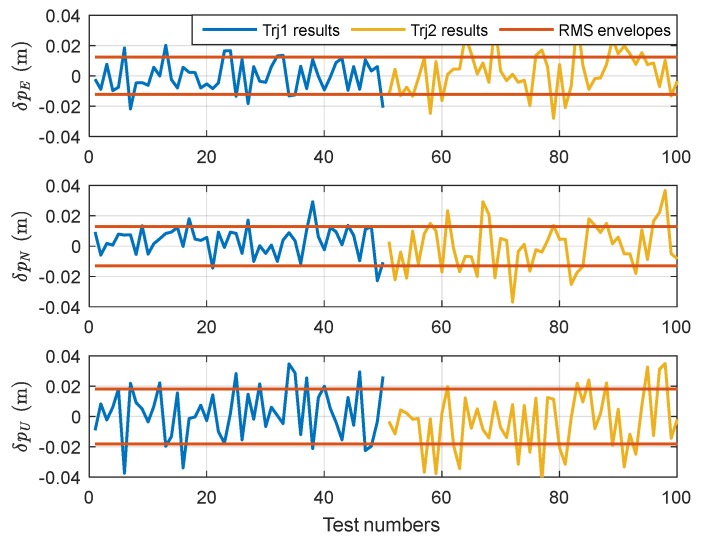
The positioning results of SINS/GPS navigation with lever arm automatic estimation.

To evaluate lever arm estimation accuracy and positioning accuracy, we carried out 100 Monte-Carlo simulations, 50 times for each trajectory. The final lever arm estimation results at the end of every test are shown in Figure 5, and Figure 6 shows the final positioning results. In these Figures, the terms “Trj1” is the abbreviation for Trajectory 1, “Trj2” is the abbreviation for Trajectory 2, and “RMS” are the envelopes of root mean square error statistics.

.

As shown in Figure 5 and Figure 6, the final estimation accuracy for the equivalent dynamic lever arm $L_{IG}^{b}$ is [0.0104; 0.0110; 0.0178] m (RMS), and the positioning accuracy is [0.0123; 0.0128; 0.0181] m (RMS), which is nearly the same as the positioning accuracy of CDGPS listed in Table 2. 

In manual measurements, the lever arms can be measured with an accuracy of 1 mm level by a laser total station instrument [17], if the phase center of the GPS antenna, the sensing center of the IMU, and the rotating center of the ISP are all marked out without error. It is true that the automatic estimation method presented here cannot accomplish such accuracy, which is restricted by the accuracy of CDGPS. However, in many practical situations, it is difficult to accurately locate these three centers, especially for the IMU. As the sensitive centers of the three-axis accelerometers do not coincide, the IMU actually have a virtual sensing center determined by the calibration algorithm of the inner arms [36], and the calibration accuracy is greatly influenced by the accuracy of inertial sensors. If the sensing centers cannot be marked out physically, the accuracy of manual measuring cannot be guaranteed.

**Figure 7:**
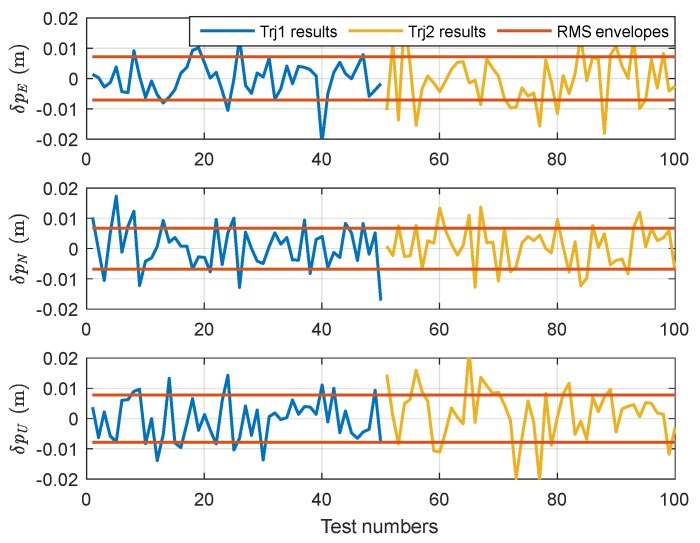
The positioning results of SINS/GPS navigation with lever arm manual measuring.

The purpose of the lever arm compensation is to obtain a higher positioning accuracy of SINS/GPS integration. Assuming that the lever arm has been manually measured with an accuracy of 1 mm, under the same simulation conditions, we obtain the positioning results of the SINS/GPS integration as shown in Figure 7.

The positioning accuracy of Figure 7 is [0.0071; 0.0068; 0.0078] m (RMS), which is slightly higher than that of the automatic estimation method. Even so, compared with the state-of-art product, POS AV610 [4], which can accomplish typical positioning accuracy of 0.02 m (horizontal)/0.05 m (vertical) by post-processing, the accuracy of the proposed automatic estimation method is still acceptable. Thus, the automatic estimation method is an effective alternative approach for certain situations, in which the measuring conditions are not available or the dynamic lever arm cannot be measured accurately and efficiently.

### 4.3. Error Distribution Simulation Results

**Figure 8:**
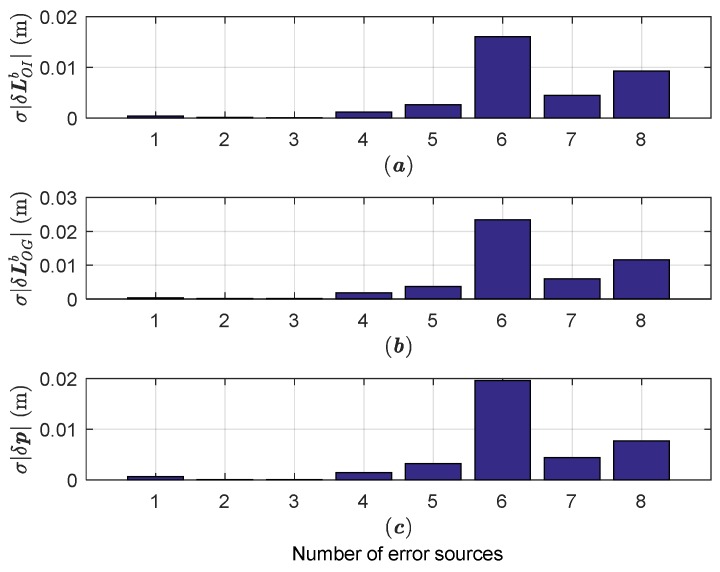
The error distribution budgets. (**a**) The estimation error of the norm of the lever arm $L_{OI}^{b}$; (**b**) The estimation error of the norm of the lever arm $L_{OG}^{b_{c}}$; (**c**) The estimation error of the norm of positioning errors.

The error distribution simulation tests are devoted to determining which error sources primarily affect the estimation accuracies of specific parameters. For sake of simplicity, the associated error sources in full-order model are classified into eight categories, as shown in Table 3, with the same RMS values as Table 2. The noteworthy parameters are chosen as the norm of lever arm $L_{OI}^{b}$, with RMS error denoted as $\sigma |L_{OI}^{b}|$; the norm of lever arm $L_{OG}^{b_{c}}$ with RMS error denoted as $\sigma |L_{OG}^{b_{c}}|$; and the norm of positioning error with RMS error denoted as σ|δp|. Under the Trajectory 1, the effects of each category on the estimation accuracies are illustrated in Figure 8.

As shown in Figure 8, the initial errors and process noises have little effect on the positioning and lever arm estimation accuracy, while the final positioning accuracy are mainly determined by the CDGPS error, consistent with the previous analysis. In addition, the time-asynchrony error and ISP encoder error have important influences on the final accuracy, and thus should be treated seriously. We note that the results of Figure 8 are based on input error values shown in Table 2. According to the linear error model, if the input errors expand *n* times, their effects will also increase *n* times.

## 5. Conclusions

The ISP equipped in a POS results in a variable lever arm, making it difficult to compensate. Instead of inefficient manual measurement strategies, our proposed method uses the encoder data from ISP gimbals to decompose the dynamic lever arm into two constant lever arms to be estimated automatically. Based on the established 21-dimensional state Kalman filter, our error analysis and simulation tests provide some meaningful conclusions. First, rotation of the ISP and an attitude change of the vehicle are necessary to determine the dynamic lever arm. Second, encoder errors of the ISP have a limited effect on estimation accuracy, so that general turntables can be accepted if the lever arm $L_{OG}^{b_{c}}$ is small enough. Third, precise time compensation is necessitated in both the lever arm estimation method and the manual measuring method. Last, the estimation accuracy of the dynamic lever arm mainly depends on the positioning accuracy of the CDGPS. Therefore, the proposed method can be used as an effective substitute for manual measurement with acceptable accuracy.

## Figures and Tables

**Table 1:** The design of simulation trajectories.

Steps	Start Time	Trajectory 1	Trajectory 2
Step 1	0 s	Keep stationary 30 s	Keep stationary 30 s
Step 2	30 s	Rotate inertially stabilized platform (ISP) heading gimbal 90°	Rotate vehicular heading 90°
	60 s	Rotate ISP roll gimbal 30°	Rotate vehicular roll 30°
Step 3	90 s	Rotate vehicular heading 90°	Rotate ISP heading gimbal 90°
	120 s	Rotate vehicular roll 30°	Rotate ISP roll gimbal 30°
Step 4	124 s	Keep stationary to 150 s	Keep stationary to 150 s

**Table 2:** Specifications of the sensors.

Error Types	Error Sources	Value (Root Mean Square, RMS)
Initial Errors	Initial attitude errors	[0.01; 0.01; 0.1]°
	Initial velocity errors	[0.1; 0.1; 0.1] m/s
	Initial position errors	[5; 5; 5] m
	Gyro biases	0.01°/h
	Accelerometer biases	30 μg
	Scale factor errors of gyros	3 ppm
	quadrature errors of gyros	3 arc second
	Scale factor errors of accelerometers	20 ppm
	quadrature errors of accelerometers	3 arc second
Process noises	Gyro random walk	0.001°/√h
	Accelerometer random walk	5 μg/√Hz
Measurement noises	Positioning errors of CDGPS	0.01 m (horizontal); 0.015 m (vertical)
	Encoder measurement noise	0.05°
	Time-asynchrony noises	0.1 ms

**Table 3:** Error classification.

Number	Error Combinations	Error Sources
1	Initial SINS errors	1–15, 22–39 elements of state vector in (A3)
2	Initial lever arm $L_{OI}^{b}$	16–18 elements of state vector in (A3)
3	Initial lever arm $L_{OG}^{b_{c}}$	19–21 elements of state vector in (A3)
4	Gyro random walk	Process noises $\varepsilon_{w}^{b}$
5	Accelerometer random walk	Process noises $\nabla_{w}^{b}$
6	Positioning errors of CDGPS	Measurement noises $w_{gp}$
7	Encoder measuring noises	Measurement noises $w_{\mu_{c}}$
8	Time-asynchrony noise	Measurement noise $w_{dt}$

## Appendix A

In the real-world model, besides the attitude, velocity, position, and lever arm errors listed in (13), the measurement error models of the gyros and accelerometers are extended as follows:

(A1) $$\delta {\tilde{\omega }}_{ib}^{b}=\delta K_{g}\omega_{ib}^{b}+\varepsilon^{b}=\left[\begin{array}{ccc} \delta K_{gxx} & \delta K_{gxy} & \delta K_{gxz}\\ \delta K_{gyx} & \delta K_{gyy} & \delta K_{gyz}\\ \delta K_{gzx} & \delta K_{gzy} & \delta K_{gzz} \end{array}\right]\left[\begin{array}{c} \omega_{ib}^{bx}\\ \omega_{ib}^{by}\\ \omega_{ib}^{bz} \end{array}\right]+\varepsilon^{b}+\varepsilon_{w}^{b},$$

(A2) $$\delta {\tilde{f}}_{}^{b}=\delta K_{a}f_{}^{b}+\nabla^{b}=\left[\begin{array}{ccc} \delta K_{axx} & \delta K_{axy} & \delta Ka_{gxz}\\ \delta K_{ayx} & \delta K_{ayy} & \delta K_{ayz}\\ \delta K_{azx} & \delta K_{azy} & \delta K_{azz} \end{array}\right]\left[\begin{array}{c} f_{x}\\ f_{y}\\ f_{z} \end{array}\right]+\nabla^{b}+\nabla_{w}^{b},$$

where $\delta K_{g}$ and $\delta K_{a}$ are error transfer matrices of the gyros and accelerometers, in which the diagonal elements denote the scale factor errors, while the off-diagonal elements express the quadrature errors, respectively; $\varepsilon^{b}$ and $\nabla^{b}$ are the gyro and accelerometer biases; $\varepsilon_{w}^{b}$ and $\nabla_{w}^{b}$ denote the random walk noises of gyros and accelerometers.

In summary, the 39-dimensional state error vector in the real-world model is formed as follows:

(A3) $$\begin{array}{l} x_{r}\left(t\right)=[\begin{array}{ccccccc} {\left(\phi^{n}\right)}^{T} & {\left(\delta v_{}^{n}\right)}^{T} & {\left(\delta p_{I}\right)}^{T} & {\left(\varepsilon^{b}\right)}^{T} & {\left(\nabla^{b}\right)}^{T} & {\left(L_{OI}^{b}\right)}^{T} & {\left(L_{OG}^{b_{c}}\right)}^{T} \end{array}\\ {\begin{array}{cccccc} \delta K_{g}(:,1) & \delta K_{g}(:,2) & \delta K_{g}(:,3) & \delta K_{a}(:,1) & \delta K_{a}(:,2) & \delta K_{a}(:,3) \end{array}]}^{T} \end{array}$$

where $x_{r}\left(t\right)$ denotes the state vector in the real-world model, and δK(:,i) represents the ith column vector of matrix $\delta K_{g}$ or $\delta K_{a}$.

In addition, the system driving noise of the real-world model includes the inertial sensor noises $\varepsilon_{w}^{b}$ and $\nabla_{w}^{b}$, while the measurement noise contains the GPS positioning noise, the ISP encoder noise, and the time-asynchrony noise.

## Appendix B

Based on the real world model, the state vector of the full-order filter is selected as:

(A4) $$x^{*}(t)=x_{r}\left(t\right)$$

where the superscript “*” denotes the matrix involved in full-order filter model.

The accompanying state equation of the full-order filter is designed as:

(A5) $${\dot{x}}^{*}(t)=\left[\begin{array}{cccc} F_{I} & 0_{15\times 6} & F_{13} & F_{14}\\ 0_{24\times 15} & 0_{24\times 6} & 0_{24\times 9} & 0_{24\times 9} \end{array}\right]x^{*}(t)+\left[\begin{array}{c} -C_{b}^{n}\varepsilon_{w}^{b}\\ {{}_{}}_{}C_{b}^{n}\nabla_{w}^{b}\\ 0_{33\times 1} \end{array}\right]$$

where:

$$F_{13}=\left[\begin{array}{ccc} -C_{b}^{n}(\omega_{ib}^{bx}I_{3}) & -C_{b}^{n}(\omega_{ib}^{by}I_{3}) & -C_{b}^{n}(\omega_{ib}^{bz}I_{3})\\ 0_{12\times 3} & 0_{12\times 3} & 0_{12\times 3} \end{array}\right],$$

$$F_{14}=\left[\begin{array}{ccc} 0_{12\times 3} & 0_{12\times 3} & 0_{12\times 3}\\ C_{b}^{n}(f_{x}I_{3}) & C_{b}^{n}(f_{y}I_{3}) & C_{b}^{n}(f_{z}I_{3}) \end{array}\right].$$

Accordingly, the position measurement equation of the full-order design filter is:

(A6) $$z^{*}=H_{p}^{*}x^{*}(t)+w_{gp}+w_{\mu_{c}}+w_{dt}$$

in which:

(A7) $$H_{p}^{*}=\left[\begin{array}{cc} H_{p}^{} & 0_{18\times 3} \end{array}\right],$$

where $w_{gp}$ represents the positioning noise of the GPS; $w_{\mu_{c}}$ and $w_{dt}$ are the position noises caused by encoder measuring error and time-asynchrony error, with the covariance as:

(A8) $$R_{\mu_{c}}=\mathrm{diag}{\left(\left[M_{P}C_{b_{c}}^{n}\left(L_{OG}^{b_{c}}\times \right)\sigma_{\mu_{c}}^{}\right]\right)}^{2}$$

(A9) $$R_{dt}=\mathrm{diag}{\left(M_{P}v^{n}\sigma_{dt}^{}\right)}^{2}$$

in which $\sigma_{\mu_{c}}^{}$ and $\sigma_{dt}^{}$ are the mean square deviation of the encoder measurement noise and time-asynchrony noise.

## Appendix C

Referring to Table 2, the initial state $x_{0}$, initial covariance matrix $P_{0}$, and system noise variance matrix $Q_{k}$ needed by the state equation of the reduced-order filter are set as:

(A10) $$x_{0}=0_{21\times 1},$$

(A11) $$P_{0}=\mathrm{diag}{\left(\begin{array}{l} {}[0.01^{\circ };0.01^{\circ };0.1^{\circ };\;0.1\;m/s;\;0.1\;m/s;\;0.1\;m/s;\;5\;m;5\;m;5\;m;\;0.01^{\circ }/h;0.01^{\circ }/h;0.01^{\circ }/h;\\ 30\;\mu g;30\;\mu g;30\;\mu g;0.5\;m;0.5\;m;0.5\;m;2\;m;2\;m;2\;m]\times 3 \end{array}\right)}^{2},$$

(A12) $$Q_{k}=\mathrm{diag}{\left([5\;\mu g/\sqrt{Hz};\;5\;\mu g/\sqrt{\mathrm{Hz}};5\;\mu g/\sqrt{\mathrm{Hz}};0.001^{\circ }/\sqrt{h};\;0.001^{\circ }/\sqrt{h};\;0.001^{\circ }/\sqrt{h};\;0_{15\times 1}]\times 3\right)}^{2}t_{s},$$

where $t_{s}$ denotes the state propagation cycle, set as 0.01 s and equal to the SINS solution cycle. The units given above are for easy interpretation, and should be changed to *S*I in practical application.

According to the specifications of CDGPS, the position measurement covariance matrix of the reduced-order filter is set as:

(A13) $$R_{k}=\mathrm{diag}{\left([0.01;\;0.01;\;0.015]m\right)}^{2}$$
